# Food‐specific IgA levels in esophageal biopsies are not sufficiently high to predict food triggers in eosinophilic esophagitis

**DOI:** 10.1002/iid3.1029

**Published:** 2023-09-27

**Authors:** Lior Abramson, Johanna M. Smeekens, Michael D. Kulis, Evan S. Dellon

**Affiliations:** ^1^ Division of Gastroenterology and Hepatology, Department of Medicine, Center for Esophageal Diseases and Swallowing University of North Carolina Chapel Hill North Carolina USA; ^2^ Division of Allergy and Immunology, Department of Pediatrics UNC School of Medicine Chapel Hill North Carolina USA

**Keywords:** elimination diet, eosinophilic esophagitis, esophageal biopsy, food trigger, IgA

## Abstract

**Background:**

Eosinophilic esophagitis (EoE) is an immune‐mediated disease, characterized by Th2‐type inflammation linked to specific foods. No currently available allergy tests reliably identify food triggers in EoE, leading to empiric dietary elimination strategies. Recently, milk‐ and wheat‐specific IgA in esophageal brushings were linked to clinical food triggers. In this study, we aimed to determine whether food‐specific IgA from esophageal biopsies is associated with known food triggers.

**Methods:**

A prior cohort of 21 patients (median age 39 years) with confirmed EoE underwent empirical elimination diets and subsequent reintroduction of foods to determine triggers. Archived baseline biopsies were used to quantify levels of peanut‐, milk‐, soy‐, egg‐, wheat‐specific and total IgA by enzyme‐linked immunosorbent assay.

**Results:**

Overall, 13 patients (62%) responded to the dietary elimination as determined by histology (<15 eos/hpf), with milk and egg being the most common triggers. Biopsies had varying amounts of total IgA, while food‐specific IgA was only detectable in 48 of 105 (46%) samples. Food‐specific IgA was normalized to total IgA for each sample and stratified by whether a food was a known trigger. For all foods tested, there were no significant differences in IgA between positive and negative triggers.

**Conclusions:**

Food‐specific IgA in esophageal biopsies was not associated with previously identified food triggers in this cohort. Future studies comparing food‐specific IgA in esophageal brushings, mucous scrapings, and biopsies from patients with known triggers will be critical to determining whether food‐specific IgA may serve as a biomarker for identification of EoE triggers.

## Graphical abstract

No currently available allergy tests reliably identify food triggers in eosinophilic esophagitis, so we aimed to determine whether food‐specific IgA from esophageal biopsies is associated with known food triggers. Biopsies had varying amounts of total IgA, while food‐specific IgA was only detectable in 48 out of 105 (46%) samples, and for all foods tested, there were no significant differences in IgA between positive and negative triggers. Therefore, food‐specific IgA in esophageal biopsies was not associated with previously identified food triggers in this cohort.

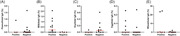

## 1 INTRODUCTION

Eosinophilic esophagitis (EoE) is a chronic, immune‐mediated disease of the esophagus characterized by symptoms of esophageal dysfunction, ≥15 eosinophils per high‐power field in an esophageal mucosal biopsy, and the absence of secondary causes of eosinophilia.^1^ Whereas EoE used to be a case‐reportable entity, it is increasingly recognized as a leading cause of upper gastrointestinal (GI) symptoms.^2^, ^3^ EoE incidence is estimated to be between 5 and 10 cases per 100,000 and prevalence is estimated at 34.4 cases per 100,000.^4^ Population‐based studies suggest that EoE incidence is increasing beyond what can be explained by improved disease recognition,^5^ and may be driven by environmental factors.^6^ Correspondingly, the proposed model of EoE links exposure to a food trigger with Th2‐mediated inflammation, leading to loss of epithelial barrier integrity and deposition of inflammatory cells in the esophageal mucosa.^7^ As such, it is postulated that the development of EoE needs either a genetic predisposition to Th2 overactivity (clinically known as an atopic state) or an acquired or intrinsic esophageal barrier defect, as well as an environmental exposure to a trigger.^8^

As EoE is triggered by foods in most people, elimination diets have become an important guideline recommended for first‐line treatment.^9^, ^10^ Since EoE is not IgE‐mediated, traditional allergy tests, such as skin prick tests and IgE quantification, are not reliable to predict EoE food triggers.^11^, ^12^ Consequently, targeted diet elimination strategies guided by conventional allergy testing have low efficacy in EoE.^13^ Empiric dietary elimination, on the other hand, has been shown to induce histologic remission in both children and adults and is the current mainstay of nonpharmacologic EoE treatment.^10^, ^14^ Although several approaches to empiric dietary elimination exist, they are all quite restrictive initially and require a time‐intensive, systematic reintroduction of possible trigger foods. Given the challenges posed by empiric elimination diets coupled with increasing prevalence of EoE, there is a growing need for the identification of effective biomarkers for targeted dietary elimination.

Our group has previously developed approaches to quantify food‐specific and total IgG4 in esophageal biopsies, and CD4^+^ T cell proliferation in peripheral blood mononuclear cells (PBMCs) from patients with active EoE.^15^ These assays had higher accuracy compared to previous allergy test methods. Specifically, accuracy rates with these assays ranged from 53% to 75%, substantially higher than those reported for skin prick testing alone, which were <15%.^11^, ^12^, ^16^ Another study demonstrated that elevated food‐specific IgA and IgG4 in esophageal brushings were associated with food triggers in patients with active EoE.^17^ Specifically, participants with wheat or dairy triggers had significantly higher wheat‐ or casein‐IgA, respectively, compared to participants that did not have those food triggers. These results suggest that food‐specific IgA may be effective for determining wheat and dairy triggers. Since EoE is diagnosed via esophageal biopsies, replicating this finding for a broader range of foods in mucosal samples is an important next step. Here, by quantifying peanut‐, milk‐, soy‐, egg‐, and wheat‐IgA in esophageal biopsies from EoE patients with known triggers, we aimed to determine whether food‐specific IgA could be used to predict response to dietary elimination.

## 2 METHODS

### 2.1 Clinical study population

We performed a secondary analysis of a prospective cohort study, the details of which have been previously reported.^15^ In that study, adults 18 years or older with a confirmed diagnosis of EoE and banked esophageal biopsies were enrolled at University of North Carolina. There were two parts of that parent study. In the first part, all patients were treated with six‐food elimination (SFED) and responders had food triggers identified with standard food reintroduction protocols. In the second part, a food elimination diet based on IgG4 and T‐cell stimulation results was implemented (based on data and thresholds determined in the first part), and responders also had food triggers confirmed via food reintroduction. For inclusion in the present study, the patients had to have either (1) histologic response to diet elimination therapy (either SFED or the IgG4/T‐cel‐based diet) with reintroduction of food triggers such that food triggers were known, or (2) nonresponse to SFED. We then utilized baseline (pretreatment—before the initial of diet elimination therapy) esophageal biopsy samples that had been stored at −80°C. We matched these with characteristics including patient demographics, EoE clinical features, and previously identified food triggers. Histologic response was defined as a posttreatment peak esophageal eosinophil count of <15 eos/hpf after at least 6–8 weeks of dietary elimination.^18^, ^19^ A food trigger was defined by recurrent inflammation (peak eosinophil count >15 eos/hpf after at least 6–8 weeks of reintroduction of that single food).^10^, ^20^, ^21^ This study was approved by the UNC Institutional Review Board (#15‐2719).

### 2.2 Measurements of food‐specific IgA and analyses

Biopsy samples were homogenized in protease inhibitors, as previously reported.^15^, ^22^ Total protein concentrations were quantified by bicinchoninic acid (BCA) protein assay (Thermo Fisher Scientific) and all samples were normalized to 100 µg/mL total protein before IgA enzyme‐linked immunosorbent assays (ELISAs). Food protein extracts were prepared from peanut flour (Golden Peanut), nonfat dry milk powder (The Milky Whey), soy flour (Honeyville), egg white powder (Deb El Foods), and wheat flour (Honeyville), as previously described.^15^, ^23^ For food‐specific IgA ELISAs, 96‐well plates were coated with 20 µg/mL peanut, milk, soy, egg, or wheat flour for samples or anti‐human IgA1/IgA2 (clone G18‐1, BD Biosciences) for standard curves in carbonate‐bicarbonate buffer. Plates were blocked with 2% BSA in PBS with 0.05% Tween 20. Biopsy samples were diluted 1:5 for all antigen‐specific ELISAs. Standard curves were prepared with purified human IgA (Bethyl Labs,) ranging from 0.6 to 60 ng/mL. Samples were detected with a 1:1000 dilution of anti‐human IgA‐HRP (Southern Biotech). Plates were developed using TMB (SeraCare) and subsequently stopped with 1% HCl (SeraCare), before reading plates at 450 nm using a microplate spectrophotometer (BioTek Instruments). For total IgA ELISAs, the same protocol was used with the following changes: for coating, anti‐human IgA1/IgA2 was used for samples and standard curves, and samples were diluted 1:250. IgA concentrations were calculated for each sample based on the standard curves generated, with a limit of detection of 0.005 ng/mL. We then examined the distributions of food‐specific IgA levels, and examined levels for those with and without the clinically identified food‐specific trigger. For statistical analysis, clinical data were summarized with descriptive statistics, and pre‐/posttreatment outcomes were compared among groups with paired *t* tests. For the IgA and associated data, unpaired t‐tests were performed between groups. GraphPad Prism version 9 was used to analyze all data, and a *p* value < .05 was considered significant.

## 3 RESULTS

### 3.1 Patient characteristics

Samples from 21 participants, with an average age of 39 years, were available for analysis (Table 1). Ninety percent of participants had dysphagia, while other symptoms, including heartburn, chest pain, abdominal pain, and nausea/vomiting, were less common. Seventy‐six percent of participants had an atopic condition, with the majority (67%) having allergic rhinitis. Overall, 13 patients (62%) were histologic responders (<15 eos/hpf level) to the dietary elimination. The most common triggers identified during prior clinical care were dairy (76%) and egg (50%).

**Table 1:** Clinical characteristics of the study population (*n* = 21).

	Mean or number (%)
Age (mean years ± SD)	39.0 ± 13.3
Female (*n*, %)	14 (67)
White (*n*, %)	20 (95)
Symptoms (*n*, %)	
Dysphagia	19 (90)
Heartburn	11 (52)
Chest pain	5 (24)
Abdominal pain	3 (14)
Nausea/vomiting	4 (19)
Any atopic condition (*n*, %)	16 (76)
Asthma	6 (29)
Allergic rhinitis	14 (67)
Food allergy	8 (38)

The average eosinophil count in all participants pretreatment was 71.8 eos/hpf at baseline and 27.6 eos/hpf post‐diet. Histological responders had a baseline eosinophil count of 63.1 eos/hpf and 1.8 eos/hpf post‐diet. Histologic non‐responders had a baseline eosinophil count of 81.1 eos/hpf and a post‐diet eosinophil count of 62.0 eos/hpf. There was no significant difference between baseline counts in responders and non‐responders (*p* = .16; Table 2).

**Table 2:** Histological response to diet elimination.

	Mean ± SD (eos/hpf)	Range
All Participants		
Baseline	71.8 ± 25.5	25–110
Post‐diet	27.6 ± 39.9	0–120
Histologic responders (<15 eos/hpf)		
Baseline	63.1 ± 28.5	35–110
Post‐diet	1.8 ± 2.1	0–5
Histologic nonresponders (≥15 eos/hpf)		
Baseline	81.1 ± 26.6	25–100
Post‐diet	62.0 ± 32.7	25–120

### 3.2 Food‐specific and total IgA quantities

**Figure 1:**
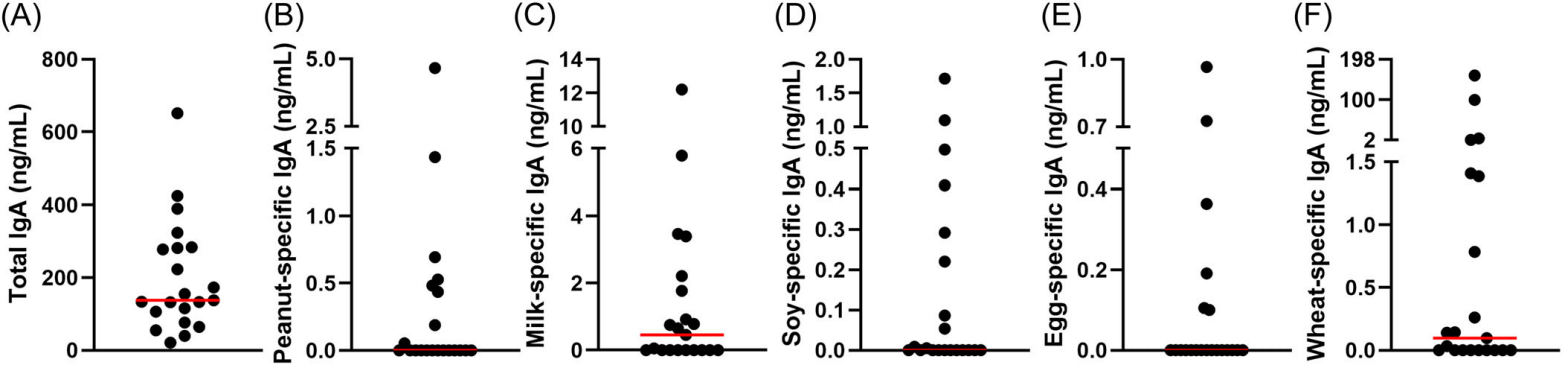
Total and antigen‐specific IgA quantities from EoE biopsy samples. (A) Total IgA, (B) peanut‐specific, (C) milk‐specific, (D) soy‐specific, (E) egg‐specific, and (F) wheat‐specific IgA. Data from individual samples are shown with red lines representing the median. EOE, eosinophilic esophagitis.

Biopsy samples had varying total IgA, ranging from 21 to 651 ng/mL, with a median of 137.5 ng/mL (Figure 1A). Food‐specific IgA was not detectable in the majority of samples, with peanut, soy, and egg having a median IgA of 0 ng/mL. Milk‐ and wheat‐specific IgA were higher in samples with median levels of 0.46 ng/mL and 0.1 ng/mL, respectively. Peanut‐specific IgA ranged from 0 to 4.7 ng/mL (Figure 1B); milk ranged from 0 to 12.2 ng/mL (Figure 1C); soy ranged from 0 to 1.7 ng/mL (Figure 1D); egg ranged from 0 to 0.97 (Figure 1E); and wheat ranged from 0 to 158.7 ng/mL (Figure 1F). Overall, food‐specific IgA was only present at levels greater than the limit of detection in 48 out of 105 (46%) samples.

### 3.3 Food‐specific to total IgA ratios based on known food triggers

**Figure 2:**
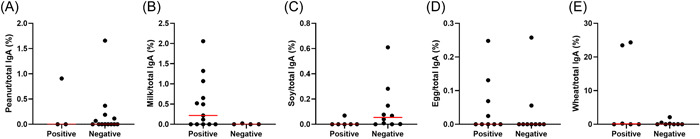
Normalized food‐specific IgA results stratified by whether food triggers were positive or negative. Peanut‐specific (A), milk‐specific (B), soy‐specific (C), egg‐specific (D), and wheat‐specific (E) to total IgA ratio (shown as a percentage) stratified by whether food triggers were positive or negative. Data from individual samples are shown with red lines representing the median.

Although food‐specific IgA quantities were very low in the majority of samples, we hypothesized that normalizing food‐specific IgA to total IgA would be more informative for assessing whether known food triggers would correlate with increased IgA quantities. Each food‐specific IgA was normalized to total IgA,^24^, ^25^, ^26^, ^27^ and stratified by whether the food was a known trigger (Figure 2A–E). For all foods, there were no significant differences in IgA between positive and negative triggers. Milk‐specific IgA showed a trend towards an increase in IgA in subjects with a known trigger; however, this did not reach statistical significance (*p* = .16). No IgA was detected in 24 out of 44 samples with a known negative trigger (54%), and similarly no IgA was detected in 21 out of 37 samples with known positive triggers (57%), demonstrating no association between positive triggers and IgA (*p* = .84).

## 4 DISCUSSION

Presently, dietary elimination is a guideline recommended for first‐line treatment option in the management of EoE.^9^, ^10^ There are two conceptual approaches to dietary elimination: targeted versus empiric. Targeted elimination strategies using conventional allergy testing have fallen out of favor due to their generally low rates of efficacy.^13^ Empiric diets, such as an elemental diet comprising of hydrolyzed amino acid formulation, and the six‐food elimination diet, are restrictive and time‐intensive for both patients and providers. These diets require sequential reintroduction of food triggers followed by endoscopic evaluation to assess response, even if less restrictive initial approaches are used.^14^, ^28^ Therefore, much work is being done to explore novel methods of targeted dietary elimination. IgA is a secreted antibody with a key role in mucosal immunity and inflammation. Although the role of IgA in allergy‐mediated processes is not well established, recent evidence points to an association between IgA levels in esophageal secretions and wheat and dairy triggers of EoE.^17^, ^29^ To further investigate these findings, we attempted to replicate the association of food‐specific IgA levels with known trigger foods in esophageal mucosal biopsies. In our cohort, however, food‐specific IgA levels in esophageal biopsies were not predictive of response to dietary elimination.

Recent work has focused on novel immunologic methods to identify food triggers. Our team previously developed a new approach investigating CD4^+^ T cell proliferation in PBMCs and food‐specific IgG4 in esophageal biopsies, which had higher success identifying trigger foods compared to previous reports with allergy tests.^15^ In a different study, milk‐specific IgG4 levels in plasma were not shown to be significantly different between EoE patients with and without milk allergy.^30^ In that same cohort, however, milk‐specific T‐helper cell proliferation was useful in predicting milk allergy. Work on the “esophageal prick test” has also examined immunologic tissue responses to stimulation.^31^ Interestingly, a comparison of allergen‐specific esophageal prick testing to skin‐prick testing and IgE levels showed no relationship between these assays, suggesting that the EoE disease process is more locally mediated than systemic.^32^ A recent study in esophageal brushings from patients with active EoE demonstrated that increased food‐specific IgA and IgG4 were associated with positive wheat and dairy triggers.^17^ Based on these results, we hypothesized that food‐specific IgA quantities from esophageal biopsies would be associated with dietary response to food‐trigger elimination in subjects with active EoE. Surprisingly, we were unable to replicate the association between esophageal IgA and known food triggers. An important distinction between our work and Peterson et al., however, is the origin of our respective specimens. Whereas Peterson et al. used esophageal brushings and analyzed esophageal mucous and associated secretions,^17^ we used esophageal tissue samples (hypothesizing that the mucous layer may remain intact in these flash‐frozen specimens). Given that IgA is a secreted antibody that is not typically present in the esophageal tissue itself, food‐specific IgA detection in brushings or mucosal scrapings may be more robust and therefore serve as a more effective predictor.^33^ This is supported by our finding that food‐specific IgA levels in esophageal mucosa were extremely low and often undetectable across both positive and negative trigger groups and across multiple allergens. Moreover, this is consistent with our previous data that food‐specific IgG4 from esophageal biopsies was associated with food triggers, since unlike IgA, IgG4 is present in the tissue in high quantities.^15^

Although we initially hypothesized that IgA quantities in tissue would be associated with dietary response, our findings support a conclusion that IgA is too low to be a useful biomarker in esophageal biopsies. We acknowledge that given the secretory nature of IgA, esophageal biopsies are not likely to be the optimal bio‐source for quantifying this specific immunoglobulin. However, given that esophageal biopsies are the mainstay for evaluation of EoE activity, these are often the most accessible sample types in clinical research on EoE, as was the case in our cohort. Moreover, this finding, taken into consideration with previously reported data on IgA, helps to further define the role of IgA in guiding dietary elimination in EoE. Accordingly, it is important to characterize the efficacy of potential biomarkers in mucosal samples specifically. Although our data does not contradict the finding of Peterson et al. that IgA levels are associated with wheat and dairy triggers, we believe there is value in establishing that IgA is not a useful biomarker in esophageal biopsies. Additional data comparing food‐specific IgA levels in esophageal brushings, mucous scrapings, and biopsies from the same patients, ideally in those with known triggers, would be helpful in determining the optimal method for sampling esophageal IgA. Novel noninvasive collection methods that rely on esophageal secretions, such as an esophageal sponge or the string test, in combination with food‐specific IgA detection assays, may be an interesting future direction.^34^, ^35^

Studies investigating targeted dietary elimination in EoE, including our own, have been limited by small sample sizes, a limited numbers of trigger foods tested, and have focused primarily on adult patients. Future work should address these limitations by enrolling subjects across multiple research centers, expanding the age of patients, and analyzing a larger subset of trigger foods. Given that our study was a secondary analysis of previously collected samples, we acknowledge the limitation that storage of specimens may have affected sample quality and ability to optimally detect food‐specific IgA levels. However, our methodology was strengthened by the rigor of the prospective parent study, sample collection, and elimination diet data from the previous trial.

In conclusion, food‐specific IgA quantified in esophageal biopsies from patients with active EoE was not associated with known food triggers. Although this contradicts previously reported data from esophageal brushings, the expected localization of IgA in the mucosal lumen compared to esophageal tissue may account for these differences. Further investigation into the role of food‐specific IgA as predictors of food triggers as well as the method for best obtaining esophageal samples for IgA quantification is warranted to develop a more accurate approach to identify food triggers in EoE.

## AUTHOR CONTRIBUTIONS

**Lior Abramson**: Conceptualization; data curation; methodology; writing—original draft; writing—review and editing. **Johanna M. Smeekens**: Conceptualization; data curation; formal analysis; methodology; writing—original draft; writing—review and editing. **Michael D. Kulis**: Conceptualization; data curation; methodology; supervision; writing—review and editing. **Evan S. Dellon**: Conceptualization; data curation; funding acquisition; methodology; supervision; writing—review and editing.

## CONFLICT OF INTEREST STATEMENT

The authors declare no conflict of interest.

## ETHICS STATEMENT

This study was approved by the UNC Institutional Review Board. Informed consent was obtained from patients who contributed samples for this study.

## Data Availability

The data sets generated for this study may be available upon request.
